# Supplementary figures and images for: Sensitivity optimisation of tuberculosis bioaerosol sampling
Source: PLoS One. 2020 Sep 3;15(9):e0238193. doi: 10.1371/journal.pone.0238193 (PMC7470324; doi:10.1371/journal.pone.0238193)

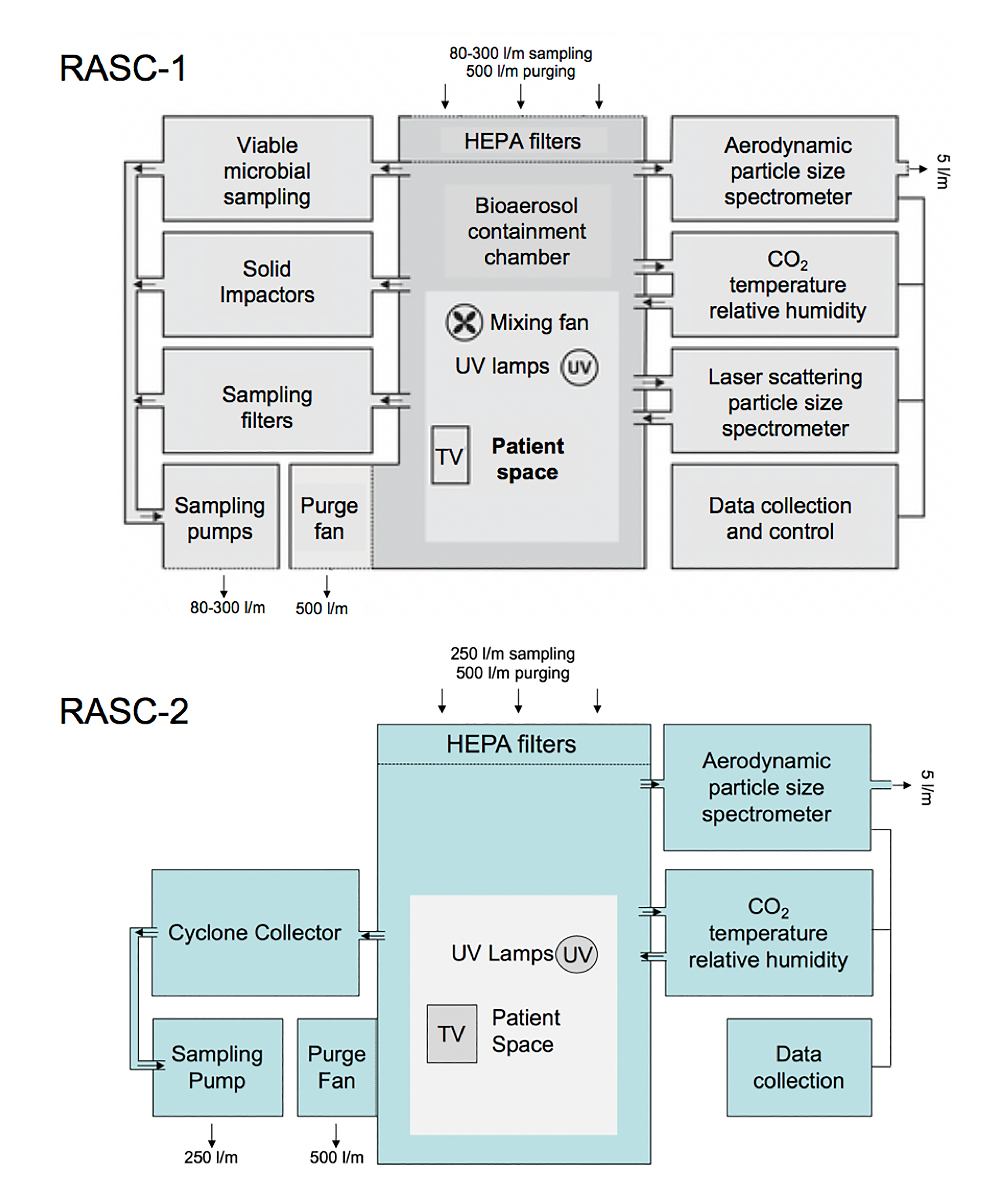

Supplement: S1 Appendix — (TIF) [file pone.0238193.s001.tif]
