# Supplementary material for: Sensitivity optimisation of tuberculosis bioaerosol sampling
Source: PLoS One. 2020 Sep 3;15(9):e0238193. doi: 10.1371/journal.pone.0238193 (PMC7470324; doi:10.1371/journal.pone.0238193)
Supplement: Attachment — Submitted filename: ResponseToReviewers_R2.docx [file pone.0238193.s003.docx]

ACADEMIC EDITOR: The crux of this paper is that an improvised air sample collection system increased the sensitivity of Mtb detection. As Rev#1 mentioned in previous round of review, this could be done without comparing the two methods, since they cannot be compared head-to-head. As can be seen from the instrumentation picture, these two air collection systems are not comparable. The parameters used to collect samples, the pressure of the unit, and the Mtb detection methods are very different. As the authors noted, since multiple parameters are changed, it is hard to define which parameter contributed to the improved sensitivity. Then, a question is "why to compare these two in the first place ?". Thus, i would suggest the authors to present only the features of the latest model (RASC-2), its design, parameters and performance. The previous publication on the RASC-1 model can be discussed in the "Discussion" section, if/when relevant. Alternatively, if the authors still want to compare these two systems head-to-head, then add a table to explicitly mention all the minute differences between the two units on every parameter, such that the reader would understand what "optimization" happened from RASC-1 to become RASC-2.

Thank you very much for your continued assessment of this paper.

Researchers have been trying to identify *Mtb* in aerosols since Chausee in 1913 with very gradual increases in the proportion of TB cases identified with *Mtb* positive aerosols over the last century. Our comparison of sampling and detection systems increased the proportion of patients with detectable airborne *Mtb* from ~40% to approaching 100% and highlights some of the specific drivers required to improve sensitivity. The lowered limit of detection is a result of the improvements in the entire system (sampler, sampling volume and detection method). Comparing sampling outputs between the two systems - in the form of exhaled air volume and bioaerosol volume (Fig. 2) – demonstrates the sampling component of this improvement. The organism recovery and detection component is demonstrated by plotting organism count per 100L of exhaled air for each patient with each system (Fig. 4). This shows the range of aerosol concentrations detected and highlights the zero-inflation problem of the less sensitive system.

We therefore feel strongly that the comparison is valid and has explanatory value. For further clarity we have added the following table to the manuscript.

| **Optimisation Parameter** | **RASC-1** | **RASC-2** | **Advantage** |
| --- | --- | --- | --- |
| **Sampler** | Primarily the Andersen impactor | Cyclone collector (Coriolis μ air sampler) | Liquid collection may improve in recovery of live *Mtb* bacilli |
| **Sampling Rate and Time Period**  **(see Fig.1)** | 28L/min for 10 minutes | 250L/min for 60 minutes | Increased exhaled air volume (estimated by CO_2_. See Fig 2A.)  Increased bioaerosol content (estimated by aerodynamic particle counting. See Fig. 2B).  Improved regulation of humidity and temperature |
| **Detection Method** | *Mtb* Culture (Middlebrook 7H10 Solid Agar) | DMN-trehalose dye | More rapid result  Retains high sensitivity (see Fig 4) Indication of viability via requirement for metabolic activity for DMN-tre dye uptake |
